# Peritumoral Small EphrinA5 Isoform Level Predicts the Postoperative Survival in Hepatocellular Carcinoma

**DOI:** 10.1371/journal.pone.0041749

**Published:** 2012-07-30

**Authors:** Tong-Hong Wang, Kwai-Fong Ng, Ta-Sen Yeh, Yu-Ling Wang, Kung-Hao Liang, Chau-Ting Yeh, Tse-Ching Chen

**Affiliations:** 1 Tissue Bank, Chang Gung Memorial Hospital, Tao-Yuan, Taiwan; 2 Department of Anatomic Pathology, Chang Gung Memorial Hospital, Chang Gung University School of Medicine, Tao-Yuan, Taiwan; 3 Department of General Surgery, Chang Gung Memorial Hospital, Chang Gung University School of Medicine, Tao-Yuan, Taiwan; 4 Department of Hepato-Gastroenterology, Liver Research Center, Chang Gung Memorial Hospital, Taipei, Taiwan; University of Navarra, Spain

## Abstract

**Background:**

EphrinA5, a member of Eph/Ephrin family, possesses two alternative isoforms, large ephrinA5 isoform (ephrinA5L) and small ephrinA5 isoform (ephrinA5S). EphrinA5L is a putative tumor suppressor in several types of human cancers. However, the role of ephrinA5S in hepato-carcinogenesis remains unclear. In this study, we evaluate the role of ephrinA5 isoforms in human hepatocellular carcinomas (HCC).

**Methodology/Principal Findings:**

A total of 142 paired HCCs and peritumoral liver tissue was examined for relative expression of ephrinA5L and ephrinA5S by using quantitative real-time polymerase chain reaction. We analyzed their expression in relation to clinical parameters, disease-free survival and overall survival. Functional assays were performed to dissect the possible underlying mechanisms. Both ephrinA5L and ephrinA5S were significantly downregulated in HCCs, as compared to those in peritumoral tissue (*p = *0.013 and 0.001). Univariate analysis demonstrated that ephrinA5S was positively correlated with old age and histological grade. In multivariate analysis, high ephrinA5S expression in peritumoral tissue had better disease-free survival (*p = *0.002) and overall survival (*p = *0.045) in patients with HCC after surgical resection. Functional analysis in HCC cell lines revealed that ephrinA5S had a more potent suppressive effect than ephrinA5L on cell proliferation (*p*<0.05) and migration (*p*<0.01). Furthermore, forced expression of both ephrinA5 isoforms in HCC cell lines significantly down-regulated epidermal growth factor receptor (EGFR) expression by promoting c-Cbl-mediated EGFR degradation.

**Conclusions/Significance:**

EphrinA5S might be a useful prognostic biomarker for HCCs after surgical resection. EphrinA5, especially ephrinA5S, acts as a tumor suppressor in hepatocarcinogenesis. Peritumoral small ephrinA5 isoform level could determine the postoperative survival in hepatocellular carcinoma.

## Introduction

Human hepatocellular carcinoma (HCC) is the most common cancer in the liver and ranks third in cancer-related deaths worldwide [1]. HCC is also the most common cause of cancer mortality in men and ranks second in women in the annual report of the Department of Health in Taiwan [2], [3]. The major risk factors are chronic hepatitis infected with hepatitis B and C viruses [4]–[5]. Other etiologies include cirrhosis, alcoholic liver disease, and aflatoxin exposure [5], [6], [7]. The multifactorial etiology may reflect the heterogeneous nature of HCC in pathogenesis. Although multiple treatment modalities are available, its prognosis remains poor [8], [9], [10]. For example, partial hepatectomy is one of the potential curative treatment modalities. However, the recurrence rate is still more than 75% for patients with resectable HCCs in long-term follow-up [11], [12]. It is therefore important to identify specific biomarkers and then to develop helpful therapeutic approaches.

Studies have reported that aberrant signaling transduction through several groups of receptor tyrosine kinase plays a pivotal role in the carcinogenesis of HCC [13], [14]. Activation of these receptors and their downstream signaling pathways lead to cell proliferation, migration, anti-apoptosis and angiogenesis in HCC [15], [16], [17]. Hence, agents that specifically block their activation and signaling cascade would be valuable for treatment of HCC [18], [19]. Therefore, understanding the signaling cascade that is involved in the progression of HCC may facilitate the development of effective diagnostic and therapeutic strategies for HCC patients.

**Figure 1:**
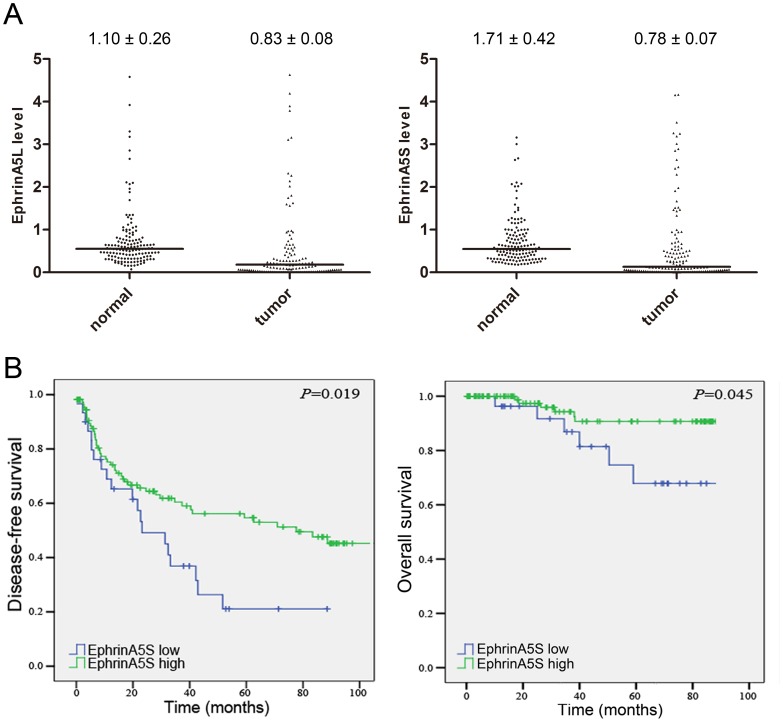
Relative expression of ephrinA5L and ephrinA5S and its relation to disease-free survival and overall survival. (A) RNA of 142 paired human HCC tissues was extracted and subjected into primer-specific real-time PCR to detect the expression of ephrinA5L and ephrinA5S. Both ephrinA5L and ephrinA5S were significantly downregulated in tumor as compared with normal tissues (*p = *0.013 and 0.001). (B) Kaplan-Meier curve for the disease-free survival and overall survival of HCC patients with high and low ephrinA5S expression. The disease-free survival and overall survival were significantly different in log-rank test with *p-*values of 0.019 and 0.045, respectively.

The Eph receptors comprise the largest family of receptor tyrosine kinases and interact with their ephrin ligands to form a bi-directional, cell-to-cell signaling communication system [20], [21], [22]. Although Eph receptors have been reported to be involved in a variety of cancers [16], [23], [24], [25], there are only a few studies addressing the genesis of HCC [26], [27], [28]. Ephrins are the ligands of Eph receptors and can be divided into two classes, ephrinA and ephrinB, differing by their modes of attachment to the plasma membrane [22], [29]. EphrinA binds to membrane by a glycosylphosphatidylinositol anchor, whereas ephrinB is a transmembrane protein. Based on the similarity of their extracellular domain sequences and the binding preference to ephrinA or ephrinB, the Eph receptor is divided into two similar classes, EphA and EphB. The receptor-ligand interactions between Eph receptors and ephrins follow a general rule that A-ligands interact preferentially with A-receptors and B-ligands with B-receptors. The only exceptions are that EphA4 and EphB2 interact with ephrinB2/3 and ephrinA5, respectively [30], [31]. The alteration of ephrin/Eph receptor expression pattern is correlated with increased invasiveness, increased metastatic potential, and consequently leads to a poor clinical outcome [25], [32], [33], [34].

EphrinA5, a member belonging to the ephrinA subclass, negatively regulates EGFR by promoting c-Cbl binding and ubiquitination in glioma [35]. EphrinA5 has two transcript isoforms, including the canonical full-length ephrinA5 (ephrinA5L) and a shorter variant (ephrinA5S), which lacks exon 4 caused by alternative splicing [36], [37]. In early studies, both ephrinA5 isoforms inhibited neurite outgrowth of dorsal root ganglia; however ephrinA5S had a less inhibitory effect on the brain during development [36]. The function of the two ephrinA5 isoforms is limitedly described in tumorigenesis. In this study, we investigate the prognostic roles of ephrinA5 isoforms in HCCs and its potential downstream regulatory mechanism.

**Table 1:** Clinical parameters of HCC patients analyzed.

Clinical parameters		Value	
Total number of patients		142	
Gender-male, n (%)		119 (83.8%)	
Age (years)		56.2±13.0	
HBsAg-positive, n (%)		108 (76.1%)	
Anti-HCV-positive, n (%)		48 (33.8%)	
Cirrhosis, n (%)		98 (69.0%)	
Alcoholism, n (%)		45 (31.7%)	
Microvascular invasion, n (%)		54 (38.0%)	
Macrovascular invasion, n (%)		37 (26.1%)	
Capsule, n (%)		97 (68.3%)	
Ascites, n (%)		16 (11.3%)	
Histology grading			
I, n (%)		4 (2.8%)	
II, n (%)		31 (21.8%)	
III, n (%)		88 (62.0%)	
IV, n (%)		19 (13.4%)	
Tumor size (diameter, cm)		5.7±13.0	
Alpha-fetoprotein (ng/mL)		5426.7±38892.2	
Albumin (g/dL)		4.0±0.6	
Bilirubin (mg/dL)		1.3±1.7	
Prothrombin time (seconds)		12.3±1.6	
Creatinine (mg/dL)		1.2±0.9	
AST (U/L)		69.7±98.9	
ALT (U/L)		88.0±147.6	
ephrinA5-L (mRNA level)		T	0.83±0.08
		N	1.10±0.26
ephrinA5-S (mRNA level)		T	0.78±0.07
		N	1.71±0.42

**Table 2:** Regression analysis of ephrinA5 small isoform (ephrinA5S) in relation to clinical parameters.

Parameters	Category	No. of patient	EphrinA5S		
			β	95% CI	P
Sex	Female	23			
	Male	119	−0.96	−0.281, 0.088	0.303
Age (years)	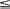 55	58			
	>55	84	0.069	0.001, 0.138	0.048*
HBsAg	Negative	34			
	Positive	108	−0.007	−0.167, 0.153	0.930
Anti-HCV	Negative	94			
	Positive	48	0.099	−0.044, 0.242	0.175
Alcoholism	Negative	97			
	Positive	45	−0.081	−0.227, 0.065	0.274
Cirrhosis	Absence	44			
	Presence	98	−0.109	−0.255, 0.038	0.145
Microvascular invasion	Absence	88			
	Presence	54	−0.048	−0.188, 0.093	0.504
Macrovascular invasion	Absence	105			
	Presence	37	0.030	−0.125, 0.185	0.704
Histology grading	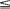 II	35			
	> II	107	0.087	0.010, 0.165	0.028*
Capsule	Absence	45			
	Presence	97	0.049	−0.098, 0.195	0.513
Largest tumor size (diameter, cm)	□ 3	51			
	>3	91	0.037	−0.105, 0.180	0.603
Ascites	Absence	126			
	Presence	16	−0.114	−0.329, 0.101	0.296
Alpha-fetoprotein (ng/mL)	□ 10.0	41			
	>10.0	101	0.012	−0.139, 0.162	0.879
Albumin (g/L)	<4.0	56			
	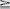 4.0	86	0.064	−0.075, 0.203	0.365
Bilirubin (mg/dL)	<0.9	44			
	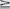 0.9	98	−0.076	−0.223, 0.071	0.311
Prothrombin time (sec)	□ 12.0	79			
	>12.0	63	0.037	−0.100, 0.175	0.591
Creatinine (mg/dL)	□ 1.0	75			
	>1.0	67	−0.080	−0.216, 0.056	0.244
AST (U/L)	□ 52	88			
	>52	54	0.072	−0.068, 0.212	0.311
ALT (U/L)	□ 111	114			
	>111	28	−0.048	−0.219, 0.123	0.578

* P<0.05.

**Table 3:** Association between ephrinA5 isoforms, clinical parameters and disease-free survival/overall survival.

Parameters	Category	No.Ofpatients	Disease-free survival (months)			Overall survival (months)		
			Mean	95% CI	P^a^	Mean	95% CI	P^a^
EphrinA5S^b^	Low ( 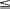 0.3)	30	35.4	23.3–47.5		85.9	72.0–99.9	
	High (>0.3)	112	73.3	61.6–85.1	0.019*	113.4	98.5–128.3	0.045*
EphrinA5L^b^	Low ( 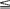 0.5)	61	47.4	37.0–57.7		93.5	84.7–102.2	
	High (>0.5)	81	72.9	59.0–86.8	0.261	109.5	91.1–127.9	0.502
Sex	Female	23	58.7	42.2–75.2		83.0	71.5–94.6	
	Male	119	65.0	53.6–76.3	0.493	111.7	98.9–124.5	0.583
Age (years)	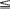 55	58	64.6	53.2–75.9		95.9	87.2–104.5	
	>55	84	56.9	44.1–69.6	0.016*	106.9	88.9–124.8	0.853
HBsAg	Negative	34	31.1	18.2–44.1		78.1	63.8–92.5	
	Positive	108	75.2	63.6–86.9	<0.001*	120.4	113.3–127.5	0.014*
Anti-HCV	Negative	94	73.4	60.7–86.1		116.8	108.0–125.7	
	Positive	48	43.8	32.1–55.5	0.057	87.6	79.4–95.8	0.655
Alcoholism	Negative	97	70.0	57.5–82.5		105.8	91.2–120.5	
	Positive	45	48.4	35.4–61.4	0.437	94.7	89.5–99.9	0.103
Cirrhosis	Absence	44	55.8	42.3–69.3		90.0	82.2–97.9	
	Presence	98	63.1	50.9–75.4	0.445	105.7	88.2–123.3	0.446
Microvascular invasion	Absence	88	74.6	61.6–87.6		105.4	88.1–122.8	
	Presence	54	44.1	32.6–55.6	0.026*	98.7	90.2–107.2	0.456
Macrovascular invasion	Absence	105	64.9	53.4–76.4		109.0	94.5–123.6	
	Presence	37	59.0	43.0–75.1	0.737	89.4	78.9–100.0	0.789
Histology grading	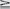 II	35	72.4	53.2–91.6		118.7	106.3–131.2	
	> II	107	52.1	43.4–60.9	0.437	87.5	81.7–93.2	0.564
Capsule	Absence	45	53.2	41.4–65.1		96.5	87.3–105.7	
	Presence	97	64.0	51.4–76.6	0.538	106.7	89.0–124.4	0.794
Largest tumor size (diameter, cm)	□3	51	79.5	63.4–95.5		120.4	110.9–129.8	
	>3	91	48.0	38.6–57.4	0.036*	86.2	79.3–93.0	0.304
Ascites	Absence	126	66.4	55.7–77.2		110.9	98.4–123.4	
	Presence	16	52.9	28.0–77.7	0.875	87.8	75.7–99.8	0.808
Alpha-fetoprotein (ng/mL)	□ 10.0	41	88.5	71.9–105.0		124.4	116.2–132.6	
	>10.0	101	45.8	36.8–54.7	0.003*	84.4	77.6–91.3	0.053
Albumin (g/L)	<4.0	56	40.2	28.8–51.6		98.3	89.4–107.3	
	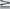 4.0	86	77.0	64.1–89.9	0.004*	106.2	88.9–123.6	0.604
Bilirubin (mg/dL)	<0.9	44	77.0	58.5–95.4		115.9	102.5–129.2	
	≧ 0.9	98	50.3	41.5–59.0	0.231	94.2	86.9–101.6	0.980
Prothrombin time (sec)	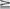 12.0	79	72.1	58.3–85.9		113.6	103.5–123.6	
	>12.0	63	48.6	37.7–59.6	0.234	97.3	89.2–105.4	0.451
Creatinine (mg/dL)	□ 1.0	75	49.7	39.8–59.6		88.2	81.8–94.5	
	>1.0	67	73.4	58.2–88.7	0.201	115.7	104.8–126.7	0.872
AST (U/L)	□ 52	88	73.6	60.8–86.5		116.0	106.7–125.3	
	>52	54	44.0	32.0–55.9	0.032*	88.8	81.3–96.2	0.959
ALT (U/L)	□ 111	114	73.3	61.9–84.7		116.4	108.3–124.5	
	>111	28	29.5	15.9–43.0	0.001*	88.2	78.5–97.9	0.818

a Kaplan-Meier analysis with log rank test.

b Relative expression of ephrinA5 mRNA assessed by real-time RT-PCR using peritumoral liver tissues.

CI: Confidence Interval,

* P<0.05.

**Table 4:** Stepwise multivariate Cox proportional hazard model for independent predictors for postoperative survival.

Factors	HR	95% CI	P
Disease-free survival			
High EphrinA5S expression	0.420	0.241–0.732	0.002
Age >55 years	1.441	1.093–1.900	0.010
HBsAg positive	0.391	0.227–0.672	0.001
Tumor size >3 cm in diameter	1.750	1.031–2.971	0.038
AFP>10 ng/mL	2.372	1.319–4.266	0.004
Alb 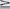 4 g/L	0.398	0.240–0.660	<0.001
Overall survival			
High EphrinA5S expression	0.342	1.014–1.023	0.045
HBsAg positive	0.278	0.093–0.830	0.022

HR: Hazard Ratio, CI: Confidence Interval.

## Results

### EphrinA5L and S are Downregulated in Human Hepatocellular Carcinomas

To elucidate the biological significance of ephrinA5 alternative isoforms in HCCs, we first examined the expression of ephrinA5L and ephrinA5S. Because no specific antibodies against ephrinA5L and ephrinA5S were available, primer-specific real-time PCR was used to study the mRNA expression of ephrinA5L and ephrinA5S in 142 paired HCCs and peritumoral liver tissue. The relative expression of ephrinA5L and ephrinA5S to reference samples are shown in Figure 1A. As compared to peritumoral liver tissue, ephrinA5L and ephrinA5S were simultaneously downregulated in 96 HCCs. Four HCCs showed ephrinA5L downregulation alone. Another 3 HCCs only had downregulated ephrinA5S. Both ephrinA5L and ephrinA5S were significantly downregulated in tumor tissue as analyzed by Wilcoxon matched pair test with *p-*values of 0.013 and 0.001, respectively. The downregulation of ephrinA5 mRNA in HCCs indicated its potential role as a tumor suppressor.

**Figure 2:**
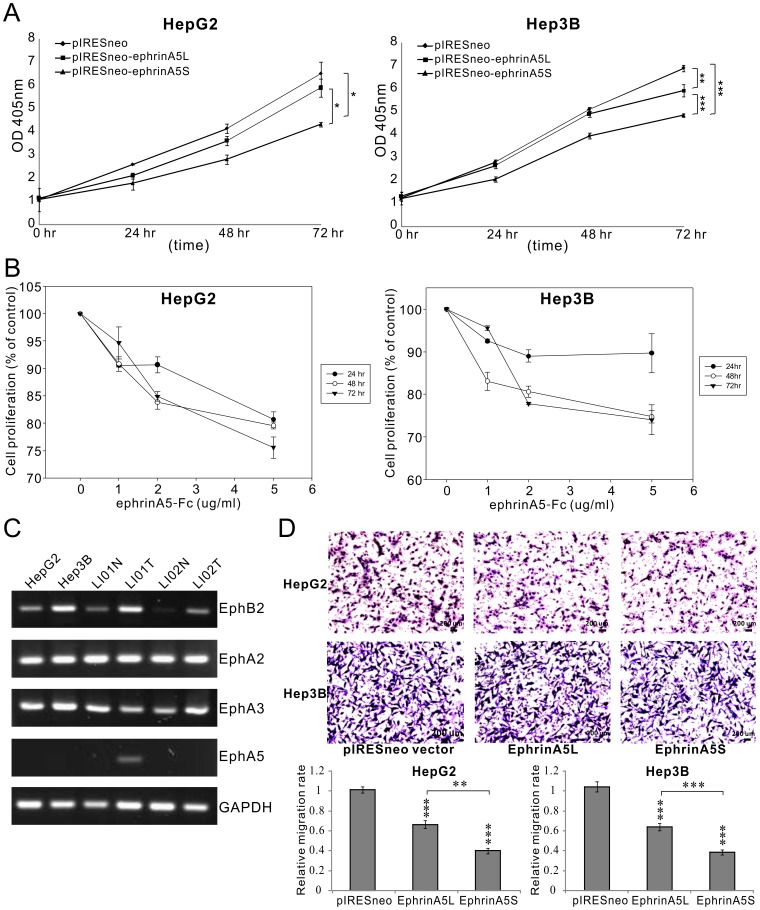
EphrinA5 isoforms suppress cell proliferation and migration. (A) Cells were transfected with 1 µg of pIRESneo-ephrinA5 isoforms or pIRESneo vector, and analyzed at the indicated times by ACP assay. Ectopic expression of ephrinA5 significantly inhibited cell growth in both HepG2 and Hep3B cells as compared to the vector control at 72 hrs. The level of significance was set at *p*<0.05 (*), *p*<0.01 (**), or *p*<0.001 (***). EphrinA5S exerted a stronger suppressive effect than ephrinA5 on both cell lines HepG2 and Hep3B. (B) Cells treated with 3 concentrations of ephrinA5-Fc were analyzed at the indicated time points by MTT assay. EphrinA5-Fc significantly reduced cell proliferation of HepG2 and Hep3B (p<0.05). (C) Expression patterns of Eph receptors in hepatoma cell lines. Primary HCCs and paratumoral tissues were analyzed by RT-PCR. EphB2, A2 and A3 expressed in both cell lines and all human HCC tissues analyzed. (D) Cell migration was compared between HepG2 and Hep3B cells transfected with pIRESneo-ephrinA5 isoforms and vector control. 5 × 10^4^ cells were plated in Transwell inserts and cultured for 24 hr in triplicates. Data were analyzed with Student’s *t-*tests. Ectopic expression of ephrinA5 significantly decreased the cell migratory ability of both cell types. The level of significance was set at *p*<0.05 (*), *p*<0.01 (**), or *p*<0.001 (***). EphrinA5S also had a stronger inhibotory effect on cell migration.

### EphrinA5S is an Independent Prognostic Predictor for Postoperative Survival

To evaluate the clinical significance of ephrinA5 isoforms, we performed linear regression analysis in the 142 HCC samples. Clinical parameters, including patients’ gender, age, HBV or HCV carrier, cirrhosis, alcoholic liver disease, degree of vascular invasion, capsule invasion, acites formation, histology grading, tumor size, alpha-fetoprotein, albumin, bilirubin, prothrombim time, creatinine, and AST/ALT for the recruited HCC cohort were summarized in table 1. In the univariate analysis, the expression of ephrinA5S positively correlated with old age (over 55 years) and histological grade. No significant association with other clinical or pathological parameters was determined (Table 2). EphrinA5L expression showed a positive correlation with serum creatinine level, and no significant difference was found in relation to other clinical parameters (Table S1). Of interest, the Kaplan-Meier survival curve with log-rank test showed that a higher ephrinA5S expression (relative expression level >0.3) in peritumoral liver tissues had a better disease-free survival and overall survival among this HCC cohort and the *p-*values were 0.019 and 0.045, respectively (Fig. 1B, Table 3). In addition, HBV surface antigen (HBsAg) was also significantly associated with better disease-free survival and overall survival, and serum albumin (Alb) was positively associated with disease-free survival (Table 3). On the contrary, elder age, microvascular invasion, larger tumor size, higher serological alpha-fetoprotein (AFP), and higher AST/ALT were associated with poor disease-free survival. To determine potential independent predictors for postoperative survival, a stepwise multivariate Cox proportional hazard model was performed. Higher ephrinA5S expression in peritumoral liver tissue, positive HBsAg and higher Alb showed a reduced lethal risk to 0.42×, 0.39× and 0.40×, respectively, whereas, elder age, larger tumor size and higher AFP exhibited 1.44×, 1.75× and 2.37× poor disease-free survival risk, respectively. For overall survival, only a high ephrinA5S expression in peritumoral liver and HBsAg reduced the lethal risk to 0.34× and 0.28×, respectively (Table 4).

**Figure 3:**
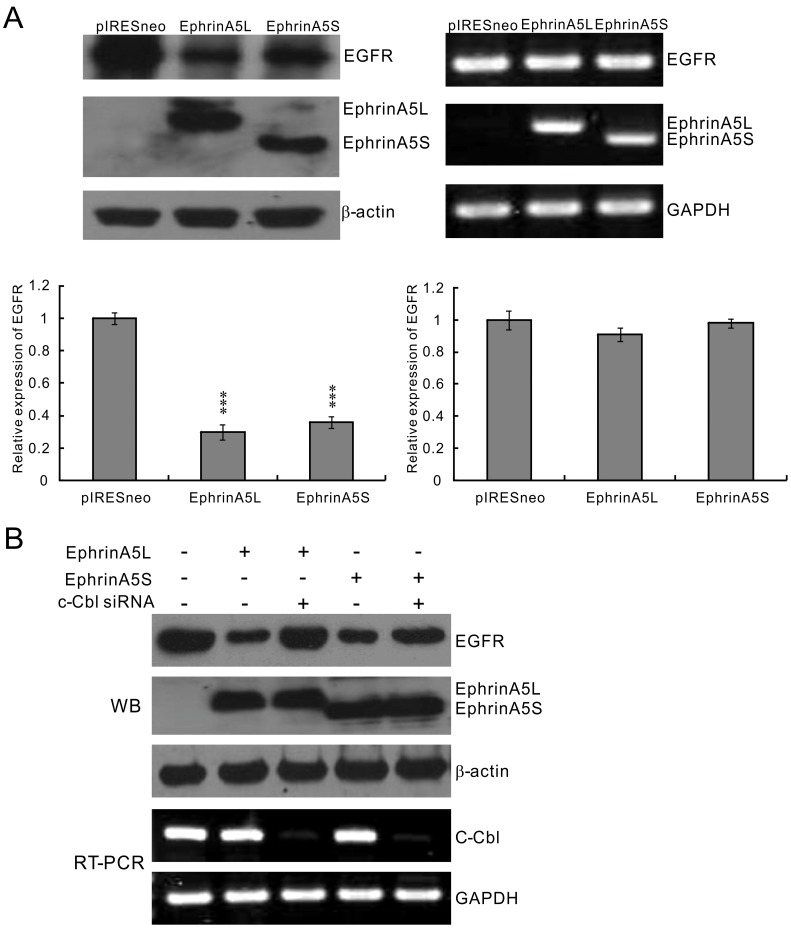
EphrinA5 isoforms suppressed EGFR expression by enhancing c-Cbl-mediated EGFR degradation. (A) Both ephrinA5L and ephrinA5S reduced EGFR protein expression level in Hep3B cells. Ectopic ephrinA5 reduced endogenous EGFR protein expression (left panel) but had no transcriptional modification of EGFR in RT-PCR (right panel). The differences were statistically significant between the treated group and untreated group. Experiments in each group were performed in triplicate. The level of significance was set at *p*<0.05 (*), *p*<0.01 (**), or *p*<0.001 (***). (B) Ectopic expresison of ephrinA5L and ephrinA5S reduced endogenous EGFR protein expression in Hep3B cells, which was rescued after c-Cbl knockdown by siRNA.

### Both ephrina5l and ephrina5s Suppress Proliferation and Migration in HCC Cell Lines

To determine the biological functions of two ephrinA5 isoforms during tumor progression, we expressed the ephrinA5 alternative variants in Hep3B and HepG2 cell lines. In an ACP assay, the overexpression of ephrinA5L and ephrinA5S significantly suppressed cell proliferation. The proliferation of both HepG2 and Hep3B was significantly inhibited up to approximately 32% by ephrinA5S at 72 hr as compared to those with pIRESneo vectors (*p* = 0.015 and *p = *0.00016, Fig. 2A). EphrinA5L only marginally suppressed Hep3B growth by 12% at 72 hr (*p* = 0.003). EphrinA5S exhibited a stronger suppressive effect than ephrinA5L on cell proliferation (*p*<0.05).

Next, we investigated the integrity of the eph receptor and ephrinA5 interaction in HCCs by analyzing the expression of EphB2, A2, A3 and A5 the preferred receptors for ephrinA5, in Hep3B and HepG2 cell lines. Except for EphA5, the expression of EphB2, A2 and A3 were detected in both cell lines. A similar eph receptor expression pattern was also found in both tumor and peritumoral liver tissues (Fig. 2C). However, EphA5 was detected in only one tumoral tissue. This result suggests that ephrinA5 is able to exhibit its tumor suppressor effect through its eph receptors in HCCs.

Next, we studied the in vitro effect of ephrinA5-Fc on cell proliferation. Hep3B and HepG2 were treated with a series of concentrations of ephrinA5-Fc for 72 hrs. A significant inhibitory effect on cell proliferation was observed in both Hep3B and HepG2 cells treated with a series of concentrations of ephrinA5-Fc (*p*<0.05; Fig. 2B). Although there was no statistical significance between different concentrations of ephrinA5-Fc, its suppressive effect had a dose-dependent trend.

Furthermore, we also examined the effect of ephrinA5 isoforms on cell migration with Transwell assays. The migratory activity of both cell lines was reduced to 35–40% and 60–65%, by ephrinA5L (*p*<0.001) and ephrinA5S transfectants (*p*<0.001), respectively, as compared to the pIRESneo vector control (Fig. 2D). These results indicate that both L and S isoforms of ephrinA5 were involved in regulating cell proliferation and migration. EphrinA5S had a more potent suppressive effect on both cell proliferation and migration.

### EphrinA5L and ephrina5s Downregulate EGFR Expression

EphrinA5L acts as a tumor suppressor by negatively regulating EGFR expression in glioma [35]. To elucidate if there was a similar regulatory mechanism for both ephrinA5 isoforms in HCC, we further examined the potential ephrinA5L and ephrinA5S suppressive effects on EGFR expression. As in Fig. 3A, overexpression of both ephrinA5 isoforms significantly reduced EGFR protein expression as compared to cells transfected with control vector pIRESneo (left panel). However, the EGFR mRNA level was not affected by any ephrinA5 variant (Fig. 3A, right panel). To address the possibility that ephrinA5 enhanced c-Cbl, the EGFR E3-ligase, to associate with EGFR and thus promote EGFR degradation, ephrinA5L and ephrinA5S was overexpressed in Hep3B cells with and without c-Cbl siRNA treatment. Hep3B cells normally expressed EGFR without detectable ephrinA5 isoforms, whereas ectopic expression of both ephrinA5L and ephrinA5S inhibited the expression of EGFR in Hep3B cells. c-Cbl siRNA treatment partially rescued the expression of EGFR in cells with ectopic expression of ephrinA5L and ephrinA5S (Fig. 3B).

## Discussion

EphrinA5 has two isoforms, the full-length ephrinA5L and the alternatively spliced ephrinA5S that lacks exon 4. The biological functions of these two isoforms have not been fully explored in carcinogenesis. EphinA5 may act as a tumor suppressor in some types of human cancers, including glioma [35], chondrosarcoma [38], and leukemia [39]. However, an oncogenetic property has also been described in some types of cancers [40], [41]. In this study, we took an advantage of a HCC cohort with long-term follow-up to evaluate the potential role of ephrinA5 isoforms in the genesis of HCC. Relative mRNA expression of ephrinA5L and ephrinA5S was analyzed by quantitative real-time PCR. Not only ephrinA5L but also ephrinA5S were significantly downregulated in HCCs as compared to those in peritumoral tissues. This result suggests that both ephrinA5 isoforms act as tumor suppressors in HCC. Univariate analysis further revealed that ephrinA5S but not ephrinA5L was positively correlated with old age (over 55 years) and histological grade. The high expression of ephrinA5S in poorly differentiated HCC might be due to passive response to active cell proliferation.

The cancer microenvironment is very complex. Tumor cells actively crosstalk with immune cells, stromal cells, endothelial cells, and even adjacent normal counterparts. Therefore, many factors, not only the tumor itself, affect the clinical prognosis and treatment effects [42], [43]. In this study, high ephrinA5S expression in peritumoral liver tissue was significantly associated with better disease-free survival and overall survival for HCC patients. The present study would be an important example that noncancerous factors affected the prognosis in HCC. Two reasons are hypothesized to explain why patients with higher ephrinA5S expression in peritumoral liver tissues had better disease-free survival and overall survival in the HCC cohort after partial hepatectomy. First, the ephrinA5S isoform was a potent tumor suppressor to prevent carcinogenesis in the remaining liver. Second, residual tumor cells after surgical resection were suppressed by enough ephrinA5S in the peritumoral tissue through the interaction of ephrin and eph receptors. For HCC, recurrence may be due to intrahepatic metastasis or the development of a second primary HCC. In this study, overexpression of ephrinA5S exerted a stronger potency than ephrinA5L on suppressing cell proliferation and migration. At the cellular level, high ephrinA5S expression could prevent malignant transformation in the individual cell. Several studies indicated that ephrinA5 interacts with more than one Eph receptor, including EphB2, A2, A3 and A5 [35], [44], [45], [46] and then modulates the signaling cascades. In this study, we found that EphB2, A2 and A3 were detected in both tumor and peritumoral liver tissues. EphrinA5-Fc significantly inhibited cell proliferation. This result suggests that the tumor-suppressive effect of ephrinA5 isoforms in HCC could be mediated by activating the downstream “forward signaling” cascade through these receptors, as demonstrated in glioma [35]. The second effect of high ephrinA5S expression in non-neoplastic cells is inhibition of the growth of residual tumor cells and prevention of deregulated proliferation in liver cells with low ephrinA5S. Therefore, high ephrinA5S expression suppressed the development of intrahepatic metastasis/recurrence and a second primary HCC.

Recent studies indicated that EGFR is frequently expressed in human hepatoma cells, and EGF is one of the mitogens that is needed for the growth of hepatoma cells [47]. Furthermore, gefitinib, an EGFR inhibitor, was demonstrated to efficiently reduce HCC cell migration and invasion [48], [49]. Hence, EGFR could be a potential therapeutic target in human HCCs. The suppressive function of ephrinA5 by modulating EGFR expression was based on the finding that ephrinA5L accelerated EGFR protein degradation by enhancing c-Cbl association with EGFR to result in EGFR ubiquitination. Overexpression of ephrinA5L further reduced colony formation and tumorigenicity in glioma cells [35]. Therefore, we explored the underlying mechanism of the suppressive effect of the ephrinA5 isoforms in the HCC cells. We found that not only ephrinA5L but also ephrinA5S suppressed EGFR expression by enhancing c-Cbl-mediated EGFR degradation. Therefore, ephrinA5S could be a new therapeutic target in clinical applications. One possibility is that up-regulation of ephrinA5S might have a synergistic effect on anti-EGFR treatment in patients with HCCs.

In conclusion, this study was the first to demonstrate that ephrinA5S functioned as a tumor suppressor by down-regulating EGFR expression in HCCs. EphrinA5S expression in peritumoral liver tissue could serve as an independent predictor for postoperative survival in patients with HCCs and as a potential therapeutic and prognostic biomarker for the treatment of HCC.

## Materials and Methods

### Patients

The study cohort consisted of 142 patients with HCCs, who underwent surgical resection at Lin-Kou Chang Gung Memorial Hospital between 2000 and 2008. Clinical and pathological characteristics were obtained from patient charts. Tumors were staged according to the seventh edition of the American Joint Committee on Cancer, and histological grade was scored according to the World Health Organization classification criteria. This study was approved by the Ethics Committee of Chang Gung Memorial hospital and written informed consent was obtained from each patient.

### Detection of ephrina5 Isoforms by Quantitative Real Time Polymerase Chain Reaction (PCR)

Total RNA was isolated by TRIzol (Invitrogen, Carlsbad, CA). After calculating the concentration of each RNA sample using a Nanodrop detector (Thermo Scientific, Wilmington, DE), RNA samples were treated with RQ1 RNase-free DNase (Promega, Madison, WI). Two micrograms of treated RNA samples was subjected to reverse transcription with SuperScript III (Invitrogen, Carlsbad, CA). Quantitative real time PCR was processed by ABI-3700 machine using a Quantifast syber green PCR kit (Qiagen, Valencia, CA), and GAPDH mRNA was used as an internal control. Real-time PCR products were also analyzed by gel electrophoresis to confirm a single PCR product. Primer sets are listed as follows.

ephrinA5L-forward: 5′-ACCAACAAATAGCTGTATGA -3′,

ephrinA5L-reverse: 5′-TCGGCTGACTCATGTACGGT -3′,

ephrinA5S -forward: 5′-ACCAACAAATGACACCGTA -3′,

ephrinA5S -reverse: 5′-CATCGCCAGGAGGAACAGTA -3′,

GAPDH-forward: 5′-AGCCTCAAGATCATCAGCAA -3′,

GAPDH-reverse: 5′-GGCATGGACTGTGGTCATGAG -3′.

### Cell Lines, siRNA and Plasmids

HepG2 and Hep3B cell lines were obtained from the American Type Culture Collection (ATCC; Manassas, VA) and cultured in DMEM medium containing 10% fetal bovine serum at 37°C in a 5% CO_2_ atmosphere. All siRNAs targeting ephrinA5 and c-Cbl were purchased from Santa Cruz Biotechnology (Santa Cruz, CA). pIRESneo-ephrinA5, CMV-based expression, and neomycin-selective plasmids containing ephrinA5 variant cDNA, were constructed by processing with an ephrinA5 cloning primer set: Forward, 5′-CATAAGCTTCCACCATGTTGCACGTGGAGATGTT-3′; reverse 5′-ATCGGATCCTGACTCATGTACGGTGTC-3′.

### Transient Transfection of pIESneo-ephrinA5 Plasmids and siRNAs

HepG2 and Hep3B cell lines were seeded in a 6-well plate at a density of 3×10^5^ cells/well overnight. The pIRESneo-ephrinA5 plasmid (0.25 µg each) or 3 µg siRNAs including si-c-Cbl or si-scrambled were added to DMEM medium with Lipofectamine™ 2000 (Invitrogen, Carlsbad, CA) for transfection. Forty-eight hours after transfection, cells were harvested and subjected to RT-PCR and western blotting to detect RNA and protein levels of ephrinA5, c-Cbl, and EGFR.

### Detection of ephrina5, Eph Receptors, c-Cbl and EGFR mRNA Levels by Conventional RT-PCR

Total RNA was extracted with TRIzol. Two micrograms of treated RNA samples was subjected to RT-PCR, and GAPDH mRNA was used as an internal control. PCR products were analyzed by gel electrophoresis. The primer sets are listed as followed.

EphA2-forward: 5′-TCAGCAGCAGCGACTTCGAGGCA-3′,

EphA2-reverse: 5′-CAGTGGCCAGGGAAGGTGCA-3′,

EphA3-forward: 5′-ATGTTTCCAGACACGGTACC-3′,

EphA3-reverse: 5′-CCATCTTCCTGAGTAGAACTGTGAGG-3′,

EphA5-forward: 5′-CCTTCTGTGGTACGACACTTG-3′,

EphA5-reverse: 5′-GGTCTGCACACTTGACAGGTG-3′,

EphB2-forward: 5′-ATGGCGCCCCTCTCCTCTGGCATCA-3′,

EphB2-reverse: 5′-ACCGCTTGGTTCTTCCCGTG-3′,

ephrinA5-forward: 5′-GCAATCCCAGATAATGGAAGAA-3′,

ephrinA5-reverse: 5′-TCGGCTGACTCATGTACGGT-3′,

c-Cbl-forward: 5′-CGCTAAAGAATAGCCCACCTTAT-3′,

c-Cbl-reverse: 5′-ATGGCCTCCAGCCCAGAACTGAT-3′,

EGFR-forward: 5′-CGGGACATAGTCAGCAGTG-3′,

EGFR-reverse: 5′-GCTGGGCACAGATGATTTTG-3′,

GAPDH-forward: 5′-TGCACCACCAACTGCTTAGC-3′,

GAPDH-reverse: 5′-GGCATGGACTGTGGTCATGAG-3′.

### Detection of ephrina5, c-Cbl and EGFR Protein Levels by Western Blot Analysis

Transfected cells were washed twice with PBS, then lysed in 200 µl of RIPA lysis buffer with protease inhibitors, and protein concentrations were determined using the Bradford Reagent (Bio-Rad, Hercules, CA). One hundred micrograms of protein from the supernatant was loaded onto an SDS-polyacrylamide gel, followed by western blot analysis to detect protein level by ephrinA5 (Abcam, Cambridge, UK), c-Cbl (Santa Cruz Biotechnology), β-actin (Novus Biologicals, Littleton, CO) and EGFR (Santa Cruz Biotechnology) antibodies. The intensity of each band was quantified by ImageQuant 5.2 (GE Healthcare, Piscataway, NJ).

### Cell Growth Determined by ACP Assay and MTT Assay

Cells transfected with ephrinA5 plasmid or shRNA were washed twice with PBS and subjected to an acid phosphatase assay [50], to detect the proliferation rate. In the MTT assay, cells were treated with 1 µg/ml, 2 µg/ml and 5 µg/ml ephrinA5-Fc (Sigma-Aldrich Inc., St Louis, MO), respectively, and analyzed at the indicated time points.

### Cell Migration Assay

The migratory ability of HepG2 and Hep3B cells was assessed by ThinCert™ Tissue Cell Culture Inserts (Greiner bio-one, Monroe, NC) with an 8 µm pore size membrane. Cells were suspended in a final concentration of 5×10^5^ cells/ml. The lower chambers were filled with 500 µl complete medium (DMEM supplemented with 10% FBS), and 100 µl of the cell suspension were loaded into each upper chamber substantially. The cultures were incubated in a humidified 5% CO_2_ incubator at 37°C for 24 hours. The cells were fixed with 500 µl methanol for 15 minutes, and then the inner surface of the upper chambers was wiped with cotton swabs to remove the nonmigrating cells. The membranes were washed with 500 µl PBS and stained with 500 µl hematoxylin for 20 minutes at room temperature. The membranes were then washed again with 500 µl PBS. The stained cells were imaged by ImagePro 6.2 software and five random fields were counted at 100× magnification.

### Statistical Analyses

A Wilcoxon matched pair test was used to analyze the significance of ephrinA5L and ephrinA5S expression in the paired HCC tissues and suppression of cell proliferation in the MTT assay. Univariate analysis was used to analyze the expression of ephrinA5 isoforms in relation to clinical parameters. Kaplan-Meier survival curves were used to see the differences between disease-free survival and overall survival, and the significance differences between the survival curves were calculated by using log-rank test. Multivariate survival analysis was carried out by using the Cox hazard regression model. Correlation coefficients between all findings were calculated using Pearson correlation. Student’s t-test was used to analyze continuous variables in the western blot, ACP and migration assays. All tests were two-sided and a p-value <0.05 was considered statistically significant. All analyses were performed by using SPSS 16.0 or Excel 2007.

## Supporting Information

Table S1 Regression analysis of EphrinA5 large isoform (ephrinA5L) in relation to clinical parameters. *: P<0.05
(DOC)
