# Supplementary material for: Peritumoral Small EphrinA5 Isoform Level Predicts the Postoperative Survival in Hepatocellular Carcinoma
Source: PLoS One. 2012 Jul 30;7(7):e41749. doi: 10.1371/journal.pone.0041749 (PMC3408466; doi:10.1371/journal.pone.0041749)
Supplement: Table S1 — Regression analysis of EphrinA5 large isoform (ephrinA5L) in relation to clinical parameters. *: P<0.05 (DOC) [file pone.0041749.s001.doc]

Table S1. Regression analysis of EphrinA5 large isoform (ephrinA5L) in relation to clinical parameters.

| Parameters | Category | No. of patient | EphrinA5L | | |
| --- | --- | --- | --- | --- | --- |
|  |  |  | β | 95% CI | P |
| Sex | Female | 23 |  |  |  |
|  | Male | 119 | 0.343 | -0.538, 1.224 | 0.443 |
| Age (years) | ≦ 55 | 58 |  |  |  |
|  | > 55 | 84 | 0.296 | -0.031, 0.623 | 0.076 |
| HBsAg | Negative | 34 |  |  |  |
|  | Positive | 108 | -0.305 | -1.066, 0.456 | 0.429 |
| Anti-HCV | Negative | 94 |  |  |  |
|  | Positive | 48 | -0.012 | -0.700, 0.675 | 0.972 |
| Alcoholism | Negative | 97 |  |  |  |
|  | Positive | 45 | 0.273 | -0.425, 0.970 | 0.441 |
| Cirrhosis | Absence | 44 |  |  |  |
|  | Presence | 98 | 0.166 | -0.537, 0.869 | 0.642 |
| Microvascular invasion | Absence | 88 |  |  |  |
|  | Presence | 54 | 0.091 | -0.579, 0.761 | 0.788 |
| Macrovascular invasion | Absence | 105 |  |  |  |
|  | Presence | 37 | 0.223 | -0.517, 0.964 | 0.552 |
| Histology grading | ≦ II | 35 |  |  |  |
|  | > II | 107 | 0.127 | -0.250, 0.504 | 0.506 |
| Capsule | Absence | 45 |  |  |  |
|  | Presence | 97 | 0.183 | -0.516, 0.881 | 0.606 |
| Largest tumor size (diameter, cm) | ≦ 3 | 51 |  |  |  |
|  | > 3 | 91 | -0.148 | -0.825, 0.530 | 0.667 |
| Ascites | Absence | 126 |  |  |  |
|  | Presence | 16 | -0.174 | -1.202, 0.855 | 0.739 |
| Alpha-fetoprotein (ng/mL) | ≦ 10.0 | 41 |  |  |  |
|  | > 10.0 | 101 | 0.112 | -0.606, 0.829 | 0.759 |
| Albumin (g/L) | < 4.0 | 56 |  |  |  |
|  | ≧ 4.0 | 86 | 0.151 | -0.514, 0.816 | 0.654 |
| Bilirubin (mg/dL) | < 0.9 | 44 |  |  |  |
|  | ≧ 0.9 | 98 | -0.295 | -0.997, 0.406 | 0.407 |
| Prothrombin time (sec) | ≦ 12.0 | 79 |  |  |  |
|  | > 12.0 | 63 | -0.065 | -0.719, 0.590 | 0.845 |
| Creatinine (mg/dL) | ≦ 1.0 | 75 |  |  |  |
|  | > 1.0 | 67 | 0.660 | 0.018, 1.303 | **0.044** |
| AST (U/L) | ≦ 52 | 88 |  |  |  |
|  | > 52 | 54 | -0.118 | -0.788, 0.552 | 0.729 |
| ALT (U/L) | ≦ 111 | 114 |  |  |  |
|  | > 111 | 28 | -0.114 | -0.931, 0.703 | 0.783 |
